# From PICU to home: a longitudinal airway safety framework for children with tracheostomies

**DOI:** 10.3389/fped.2026.1898161

**Published:** 2026-08-18

**Authors:** Zuyu Yang, Hongxing Dang

**Affiliations:** 1 Department of PICU, Children’s Hospital of Chongqing Medical University, Chongqing, China; 2 National Clinical Research Center for Children and Adolescents’ Health and Diseases, Chongqing, China; 3 Ministry of Education Key Laboratory of Child Development and Disorders, Chongqing, China; 4 Chongqing Key Laboratory of Pediatric Metabolism and Inflammatory Diseases, Chongqing, China

**Keywords:** adverse events, critically ill children, decannulation, home care, longitudinal care, pediatric tracheostomy

## Abstract

**Background:**

As survival improves among children with chronic lung disease, severe neurologic impairment, neuromuscular disorders, congenital airway anomalies, and long-term ventilator dependence, pediatric tracheostomy care has shifted from a procedure-centered issue to a sustained respiratory care challenge. Post-tracheostomy adverse events are still frequently framed as isolated complications rather than as part of a continuous airway-safety and home-transition pathway.

**Purpose:**

This article synthesizes post-tracheostomy risk across four connected domains: acute airway-threatening events, chronic cannulation-related morbidity, hospital-to-home transition, and decannulation-related functional outcomes, and develops a pediatric longitudinal airway safety framework.

**Methods:**

We conducted a structured narrative search and qualitative synthesis drawing primarily on recent pediatric guidelines, systematic and scoping reviews, multicenter studies, national database analyses, and qualitative investigations, with priority given to publications from 2010 onward.

**Results:**

Major topics addressed include hemorrhage, false passage, accidental decannulation, tube obstruction, air leak syndromes, peristomal skin injury, respiratory infection, granulation tissue, airway stenosis or malacia, persistent tracheocutaneous fistula, dysphagia, impaired phonation, and caregiver burden. The framework differs from existing guidance and reviews by using phase of care and changing dominant risk as its organizing logic, linking each phase to the care setting, surveillance priorities, responsible teams, escalation thresholds, caregiver capability, and child- and family-centered outcomes.

**Conclusion:**

A risk-stratified pathway connecting PICU care with community-based respiratory management may improve safety, reduce avoidable healthcare utilization, and better align clinical practice with the realities faced by children and families across the full trajectory of tracheostomy dependence.

## 1 Introduction

Children with tracheostomies increasingly survive critical illness and chronic respiratory disease with ongoing airway or ventilatory support needs. In infants and children with bronchopulmonary dysplasia, severe neurologic impairment, neuromuscular weakness, congenital airway anomalies, or prolonged ventilator dependence, tracheostomy initiates a care trajectory extending from the PICU through inpatient and home care to decannulation or long-term cannulation. This article examines post-tracheostomy adverse events within that longitudinal airway-safety trajectory.

Long-term management encompasses airway patency, secretion clearance, humidification, respiratory infection surveillance, respiratory support, sleep-related breathing disorders, feeding safety, and decannulation planning. These responsibilities are distributed across specialties and care settings, creating vulnerability at transitions and contributing to emergency visits, readmissions, and delayed decannulation. A care pathway coordinates sequential assessments, interventions, professional responsibilities, and escalation decisions across care settings.

Recent guidance provides complementary foundations for pediatric tracheostomy care. The American Thoracic Society (ATS) guideline addresses discharge preparation, caregiver training and supervision, home-care safety, respiratory culture practices, airway evaluation, and decannulation (1). The American Association for Respiratory Care (AARC) guideline focuses on acute-care processes, including tube selection, humidification, tube changes, skin and stoma care, and bedside coordination (2). Previous reviews summarize indications, operative techniques, complications, education, long-term management, and decannulation (3–5). Collectively, these sources define the principal domains of safe care. Their recommendations are usually organized by clinical question, care task, or complication category, leaving the links among phase-specific risk, care transitions, escalation triggers, professional responsibilities, and family outcomes less explicit.

Building on this evidence, we developed a longitudinal airway safety framework comprising four connected domains: acute airway-threatening events, chronic cannulation-related respiratory and airway morbidity, hospital-to-home transition, and decannulation-related functional outcomes. The framework organizes phase-specific risks, surveillance priorities, responsible teams, escalation thresholds, and outcomes across the trajectory from the PICU through inpatient and home care to decannulation or continued cannulation.

Caregiver capability is a clinical determinant of home safety, and is itself shaped by training, equipment availability, nighttime supervision, access to clinical support, and family resilience.

Literature search and evidence synthesis: we conducted a structured narrative search of PubMed and Embase from database inception through July 2026. Search terms combined pediatric descriptors (“child*,” “pediatric,” “infant*,” and “adolescent*”) with “tracheostom*” or “tracheotom*” and terms for complications and adverse events, tube obstruction or displacement, hemorrhage, air leak, skin or stoma injury, respiratory infection, granulation, airway stenosis or malacia, discharge and home care, caregiver burden, readmission, decannulation, swallowing, phonation, and tracheocutaneous fistula. Controlled vocabulary and free-text terms were adapted to each database. Reference lists of key guidelines, reviews, and eligible primary studies were screened for additional publications.

Titles and abstracts were screened for direct relevance to children with tracheostomies, followed by full-text assessment of potentially eligible articles. Publications were included when they addressed at least one of four domains: acute airway-threatening events, chronic cannulation-related respiratory or airway morbidity, hospital-to-home transition and caregiver capability, or decannulation and functional outcomes. Eligible evidence included pediatric guidelines, systematic or scoping reviews, multicenter or single-center cohorts, national database studies, qualitative investigations, and quality improvement studies. Mixed-age or adult evidence was used selectively when pediatric evidence was limited and the mechanism or management principle was directly applicable to children. Reports without post-tracheostomy care outcomes were excluded. Case reports were retained only for rare high-consequence complications.

Evidence sources were used according to the question addressed. Guidelines and evidence syntheses informed practice recommendations, multicenter cohorts and national databases informed event rates and outcomes, qualitative studies informed caregiver experience, and quality improvement reports informed implementation. Priority was given to publications from 2010 onward, while older studies were retained for foundational mechanisms or enduring complications. Study design, pediatric directness, consistency, and clinical applicability were considered during synthesis. Given heterogeneity in definitions, populations, and outcomes, findings were synthesized qualitatively within the four framework domains.

Framework development: the longitudinal airway safety framework was developed through literature-based synthesis and integration of guideline recommendations. Guidelines were used to identify established safety practices, while pediatric observational studies, qualitative research, and quality improvement reports informed risk distribution, care transitions, caregiver capability, and implementation priorities. Recurring findings were organized into four domains and mapped by care phase to dominant risk, surveillance priority, responsible team, escalation trigger, and intended outcome. Connections without direct comparative evidence reflect interpretive synthesis by the authors. No formal consensus process was used.

Table 1 places the proposed framework alongside the ATS and AARC guidelines and earlier syntheses of pediatric tracheostomy care, highlighting differences in scope, organizing logic, and implementation focus.

**Table 1:** Comparison of existing pediatric tracheostomy guidelines and reviews with the proposed longitudinal airway safety framework.

Source	Primary focus	Organizing logic	Contribution to the framework
ATS clinical practice guideline (1)	Discharge preparation, caregiver training and supervision, home-care safety, respiratory culture practices, airway evaluation, and decannulation.	Clinical questions and recommendations.	Provides guidance for major hospital-to-home and decannulation domains.
AARC clinical practice guideline (2)	Acute-care tube selection, humidification, tube changes, skin and stoma care, and bedside coordination.	Technical and process-based acute-care recommendations.	Provides the inpatient technical and process elements.
Previous pediatric tracheostomy reviews (3–5)	Indications, operative technique, complications, education, long-term management, and decannulation.	Clinical topic, procedural stage, or complication category.	Provides the clinical content and longitudinal concepts integrated in the framework.
Proposed longitudinal airway safety framework	Airway threats, chronic morbidity, hospital-to-home transition, and decannulation-related outcomes across the care trajectory.	Phase of care and changing dominant risk.	Connects care setting, responsible disciplines, surveillance priorities, escalation triggers, and child- and family-centered outcomes.

## 2 Changing indications and emerging tracheostomy populations

The population of children receiving tracheostomies has changed substantially over recent decades. Acute infectious causes of upper airway obstruction now account for a smaller proportion of pediatric tracheostomy indications in many centers, whereas chronic medical complexity has become increasingly prominent (5–9). Improved survival among extremely premature infants, children with complex congenital anomalies, and those with severe neurologic or neuromuscular disorders has expanded the number of children who survive critical illness but remain dependent on long-term airway or ventilatory support (1, 10, 11). This trend has transformed pediatric tracheostomy from a short-term airway procedure into a marker of chronic care needs.

This changing indication profile has direct implications for adverse-event risk. A child with an isolated surgically correctable airway lesion differs fundamentally from a child with severe hypoxic-ischemic brain injury, uncontrolled seizures, dysphagia, chronic aspiration, and ventilator dependence. In the latter child, the tracheostomy tube is only one component of a complex physiologic and caregiving system. Such children may have recurrent infections, impaired secretion clearance, poor growth, developmental delay, and frequent hospitalizations. The tracheostomy may facilitate survival and care, but it also introduces an ongoing set of risks that must be managed over months or years.

Bronchopulmonary dysplasia and chronic lung disease are particularly important contributors to tracheostomy in infancy. These children may require prolonged positive-pressure ventilation, oxygen supplementation, and careful growth optimization. Their airways are often reactive, secretions may be difficult to manage, and respiratory illnesses may trigger major setbacks. In these patients, decisions about tracheostomy, home ventilation, and eventual decannulation must account for lung growth, pulmonary hypertension risk, feeding tolerance, and family capacity for technology-dependent care (10, 11).

Neurologic impairment and neuromuscular disease constitute another major subgroup. These children are at risk because of weak cough, impaired airway protection, reduced respiratory drive, sleep-related hypoventilation, dysphagia, and recurrent aspiration. Tracheostomy may improve airway access and enable chronic ventilatory support, but it does not correct the underlying neuromotor disorder. Long-term outcomes are therefore strongly influenced by secretion burden, aspiration prevention, nutrition, rehabilitation, communication support, and caregiver resilience.

Complex airway anomalies also remain important. Children with craniofacial disorders, subglottic stenosis, tracheomalacia, laryngomalacia, vocal cord paralysis, or airway tumors may require tracheostomy for airway stabilization. Management may include serial endoscopy, airway reconstruction, assessment for sleep-related breathing disorders, ongoing surveillance, and an individualized decannulation plan.

The clinical heterogeneity of this population complicates research and care standardization. Studies of pediatric tracheostomy often include children with very different diagnoses, ages, ventilatory needs, and follow-up durations. This heterogeneity partly explains why adverse-event rates, decannulation rates, and readmission risks vary widely across reports (3, 12–19). It also underscores the need for risk stratification rather than uniform management assumptions. A child with stable upper airway obstruction and no ventilatory dependence may require a different discharge plan and follow-up intensity than an infant with BPD on home ventilation and recurrent respiratory infections.

The timing of tracheostomy in critically ill children remains an area of practice variation. Unlike adult critical care, where timing has been studied extensively, pediatric decisions are often individualized according to diagnosis, airway anatomy, anticipated ventilator course, sedation burden, surgical feasibility, and family goals. Some children benefit from earlier transition to tracheostomy because prolonged endotracheal intubation may impair comfort, delay rehabilitation, or increase laryngeal injury risk. Others may recover sufficiently to avoid tracheostomy if the clinical trajectory is favorable. This uncertainty reinforces the need for multidisciplinary decision making rather than fixed timing rules.

Ethical and family-centered decision making is particularly important in children with severe neurologic impairment or progressive disorders. In these situations, tracheostomy may prolong survival and allow discharge home, but it may also increase caregiver workload and extend technology dependence. Families may interpret tracheostomy either as a path toward recovery or as a major life-changing commitment. Clinicians should therefore discuss not only operative risk, but also daily home care, night supervision, emergency preparedness, readmission likelihood, communication limitations, feeding challenges, and realistic decannulation prospects.

Growth introduces a pediatric-specific source of variation. A tube that fits appropriately at discharge may become unsuitable as the child grows or ventilatory needs change. Tube length, curvature, flange position, and cuff requirements require periodic reassessment. Growth may increase airway caliber and respiratory reserve, improving decannulation feasibility in some children. Progressive neurologic or neuromuscular disease may instead worsen airway clearance and ventilatory dependence. Risk stratification must therefore incorporate diagnosis, airway anatomy, ventilatory dependence, secretion burden, developmental stage, family capacity, and the care environment.

## 3 Pediatric vulnerability after tracheostomy

The vulnerability of critically ill children after tracheostomy is multidimensional. It begins with anatomy. The pediatric airway is narrow, and airway resistance rises sharply as luminal diameter decreases. A small mucus plug, blood clot, or granulation lesion may therefore have a much larger physiologic effect in an infant than in an older child or adult. In addition, pediatric tracheostomy tubes have small internal diameters and are more susceptible to occlusion by thick secretions or dried material (2, 3, 6, 12). This anatomic reality makes humidification, suctioning, and secretion monitoring central safety interventions rather than routine technical details.

The early postoperative tract is another important source of risk. Before the tract matures, accidental tube displacement can make reinsertion difficult and increase the risk of false passage (2, 20–22). The risk is highest in infants and small children because the neck is short, soft tissues are mobile, and external landmarks may be less reliable. During this period, bedside personnel must know whether reinsertion should be attempted through the stoma, whether oral or nasal intubation should be prioritized, and when specialist support is required. Failure to define these steps in advance can lead to delay during a time-critical airway emergency.

Physiology further narrows the margin of safety. Infants and children with high oxygen consumption and limited functional residual capacity can desaturate rapidly during apnea, tube obstruction, or decannulation. Children with chronic lung disease, pulmonary hypertension, cardiac disease, or neuromuscular weakness may tolerate even brief airway compromise poorly. This means that early warning signs must be recognized before severe hypoxemia develops. Increased work of breathing, agitation, unexplained ventilator alarms, reduced delivered tidal volume, increased suction resistance, and subtle color change may all be clinically important.

Comorbidity modifies both event risk and event consequences. Neurologic impairment may reduce cough effectiveness and increase aspiration risk. Neuromuscular disease may impair respiratory muscle function, reducing ventilation and secretion clearance. Chronic lung disease may increase oxygen requirement and make respiratory infections more destabilizing. Airway malacia or stenosis may create dynamic obstruction that worsens during sleep, illness, or capping trials. Feeding difficulties and malnutrition may impair wound healing and immune function. These factors are often present simultaneously in critically ill children with tracheostomies.

The care environment is equally important. In the PICU, continuous monitoring, suction equipment, trained staff, and rapid escalation are generally available. On the general ward, monitoring may be less intensive and staff experience may vary. At home, the primary responders are family caregivers, sometimes supported by home nursing, telehealth, or outpatient teams. The same clinical event therefore carries different risk depending on where it occurs. A partial obstruction recognized within seconds in the PICU may be catastrophic if it occurs at home during sleep without adequate supervision.

Developmental dependence distinguishes children from adults. Young children cannot reliably describe dyspnea, tube discomfort, suctioning difficulty, or early symptoms of infection. Infants may present with nonspecific signs such as agitation, feeding intolerance, color change, or altered sleep. Older children may have fear, communication frustration, or behavioral distress related to suctioning and tube care. These developmental factors make caregiver observation and staff experience crucial components of early detection.

The hospital environment may also introduce risk through transitions between units. A child may move from the PICU to a step-down unit, then to a general ward, and finally home. Each transfer changes monitoring intensity, nurse-to-patient ratio, equipment availability, and familiarity with tracheostomy emergencies. Handoff quality becomes a safety issue. Specific information about tube size, backup tube, difficult airway status, last tube change, suction depth, ventilator settings, and emergency airway plan should be communicated consistently.

High-risk subgroups deserve particular attention. Infants younger than one year, children with ventilator dependence, those with structural airway abnormalities, children with heavy secretions, and those with prior accidental decannulation or failed decannulation attempts are more likely to require enhanced safeguards. These safeguards may include more frequent tube position checks, more intensive caregiver training, earlier outpatient reassessment, and lower thresholds for multidisciplinary review.

**Figure 1:**
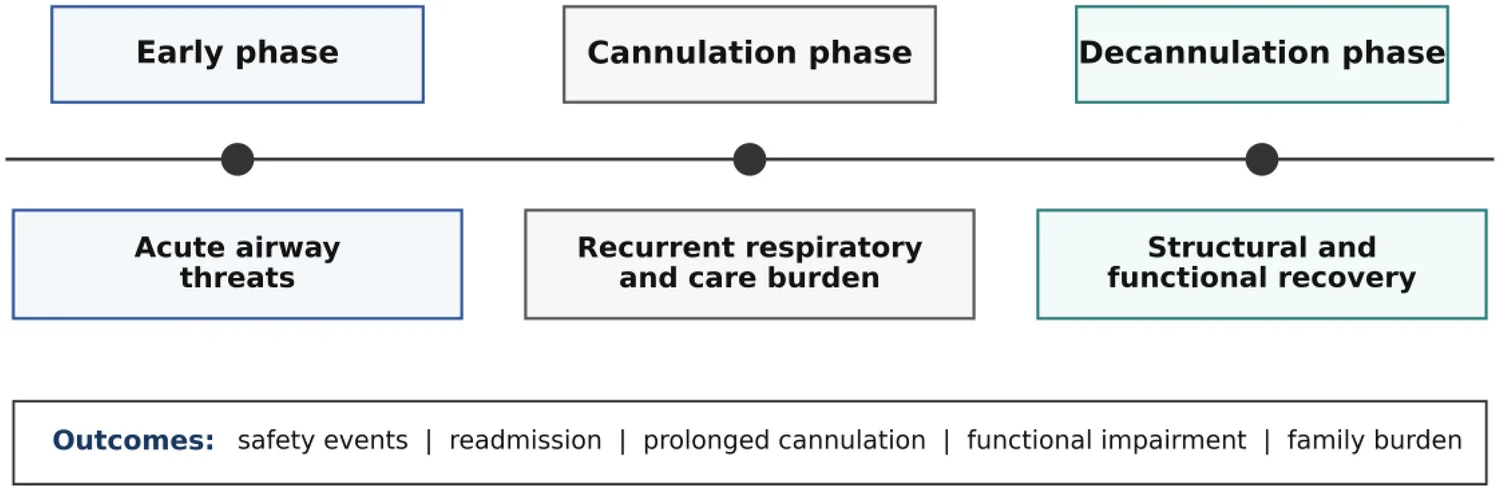
Phase-specific clinical priorities and associated outcomes across the pediatric tracheostomy care trajectory.

These vulnerabilities support a phase-based approach to pediatric tracheostomy safety. The dominant risk profile changes across the care trajectory, from early airway loss and hemorrhage to infection, skin injury, instability of home care, and decannulation uncertainty. Figure 1 shows how the dominant clinical priorities change by phase and how they relate to short-term safety events, readmission, prolonged cannulation, functional impairment, and family burden.

## 4 Acute airway-threatening events

Acute life-threatening events after pediatric tracheostomy are relatively uncommon compared with long-term care burdens, but their consequences are severe. These events include hemorrhage, air leak syndromes, accidental decannulation, false passage, and tube obstruction. They require rapid recognition, rehearsed response, and immediate access to equipment. Because the pediatric airway margin is small, the main clinical objective is to recognize early signs before respiratory failure develops.

Hemorrhage is one of the most feared complications. Minor bleeding from the stomal edge may occur early after surgery, but recurrent or unexplained bleeding should be treated as a warning sign. Catastrophic tracheoarterial fistula is rare, yet its mortality is high (16, 20, 23). Potential contributors include low tracheostomy placement, prolonged pressure from the cuff or distal tube tip, infection, abnormal vascular anatomy, and erosion from tube movement. In children, even nonmassive bleeding may obstruct the tube or airway if clots form. Therefore, bleeding should be assessed in relation to tube position, airway patency, hemodynamics, and the possibility of sentinel bleeding rather than considered only a wound issue.

Air leak syndromes, including pneumothorax, pneumomediastinum, and subcutaneous emphysema, most often occur early after tracheostomy (3, 12, 24, 25). Infants are particularly vulnerable because of high pleural apices and limited tissue depth. Warning signs include new respiratory distress, asymmetric chest movement, reduced breath sounds, increasing ventilatory pressures, neck swelling, and palpable crepitus. Prompt imaging and decompression may be required when respiratory compromise is present. The key point for pediatric clinicians is that a small air leak may have a large physiologic effect in a child with chronic lung disease or ventilator dependence.

Accidental decannulation is a defining emergency in pediatric tracheostomy care. National database and prospective cohort data show that accidental decannulation occurs most often early after surgery but remains a risk throughout hospitalization and home care (21, 22, 26). It may be triggered by turning, suctioning, agitation, coughing, loose ties, ventilator tubing traction, or poorly matched tube length. Early accidental decannulation is especially dangerous because the tract may not be mature. Reinsertion attempts may create a false passage, delaying ventilation and worsening hypoxemia.

The clinical response to accidental decannulation should be predefined. Staff and caregivers need to know the child's airway plan, tube size, backup tube size, whether oral intubation is feasible, and when to call for expert airway support. Bedside emergency kits should include the same size and smaller size tracheostomy tubes, suction catheter, lubricant, obturator, bag-valve device, oxygen source, and any child-specific instructions (2, 20, 27). Simulation can help convert written protocols into action, particularly for low-frequency events that generate panic (28, 29).

Tube obstruction is another time-critical event. It may result from thick secretions, inadequate humidification, dried crusts, blood clots, or tube kinking. Early signs include increased work of breathing, reduced chest movement, difficulty passing the suction catheter, changes in ventilator volumes or pressures, recurrent alarms, and unexplained oxygen desaturation. Complete obstruction can cause rapid cardiopulmonary collapse. Prevention depends on effective humidification, secretion assessment, suctioning technique, tube cleaning or replacement, and recognition of infection or dehydration (2, 5, 6).

**Figure 2:**
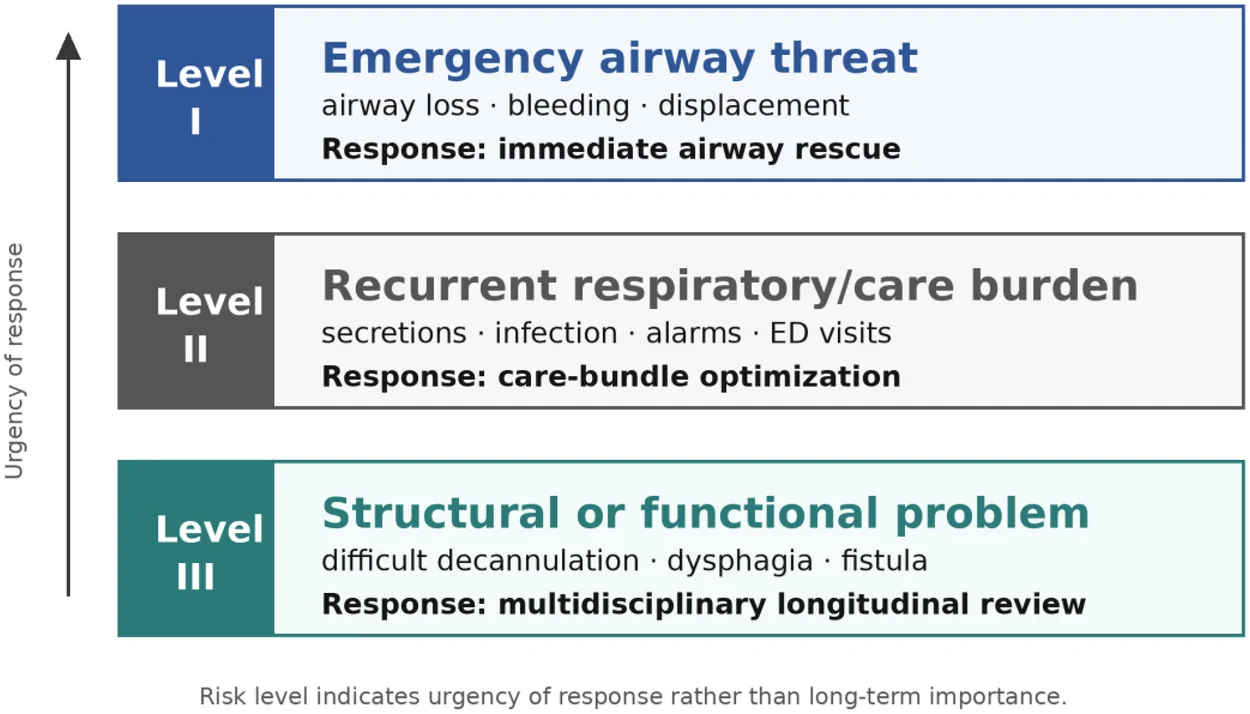
Action-oriented triage of post-tracheostomy events and clinical problems by urgency and recommended response.

Acute events are best managed through action-oriented risk stratification. Figure 2 classifies post-tracheostomy events and clinical problems by urgency and links each level to a recommended response: immediate airway rescue for acute airway threats, care-bundle optimization for recurrent respiratory and care-burden problems, and multidisciplinary longitudinal review for structural or functional problems. Table 2 summarizes major post-tracheostomy adverse events, their clinical manifestations, and key management priorities.

**Table 2:** Major adverse events after pediatric tracheostomy and key management priorities.

Adverse event	Common phase	Key pediatric-specific concern	Early recognition	Management priority
Hemorrhage	Perioperative/early; rarely late	Small-volume bleeding may obstruct a narrow airway; sentinel bleeding may precede major vascular erosion	Stomal bleeding, recurrent blood-stained secretions, clot, hemodynamic instability	Assess airway and tube position; treat significant or recurrent bleeding urgently; involve surgical airway specialists
Air leak syndrome	Early postoperative	Infants have limited reserve and may decompensate rapidly	New distress, asymmetric chest movement, crepitus, rising ventilator pressures	Prompt clinical assessment, imaging when appropriate, decompression or drainage if unstable
Accidental decannulation	Early and long-term	Immature tract increases false passage risk; home events depend on caregiver response	Tube displacement, sudden distress, inability to ventilate, loss of exhaled volume	Secure fixation, bedside emergency equipment, simulation, defined airway rescue plan
Tube obstruction	Early and long-term	Small internal tube diameter makes partial plugging clinically important	Increased work of breathing, suction resistance, alarms, reduced tidal volume, desaturation	Humidification, suctioning, tube change when needed, secretion and infection management
Peristomal skin injury	Long-term cannulation	Moisture, pressure, friction, and short neck anatomy can destabilize fixation	Erythema, maceration, erosion, drainage, pain	Daily skin assessment, moisture-wicking dressing, individualized tie tension and flange fit
Respiratory infection	Long-term cannulation/home care	Colonization complicates culture interpretation and antibiotic decisions	Fever, increased secretions, oxygen need, respiratory decline, radiographic change	Distinguish colonization from infection; culture during exacerbation; antimicrobial stewardship
Granulation tissue	Long-term cannulation/decannulation planning	May cause bleeding, obstruction, difficult tube change, or failed decannulation	Bleeding, airway obstruction, resistance during tube change, or new airway symptoms	Optimize tube fit; endoscopic assessment; treat symptomatic or obstructive lesions
Airway stenosis/malacia	Decannulation assessment	Daytime capping tolerance may not predict sleep-related hypoventilation or hypoxemia	Stridor, failed capping, nocturnal desaturation, hypoventilation, or recurrent obstructive symptoms	Airway endoscopy, individualized decannulation plan, surgical or airway intervention when needed
Persistent tracheocutaneous fistula	Post-decannulation	Longer cannulation increases persistence; affects voice, skin, cosmesis	Ongoing air leak, nonclosure, irritation, voice change	Observe early closure; surgical repair when persistent or symptomatic
Dysphagia/phonation impairment	Long-term and post-decannulation	Impacts aspiration, feeding, communication, development, and quality of life	Coughing with feeds, aspiration concern, poor growth, limited voice	Swallowing and speech assessment, nutrition and rehabilitation support, speaking valve evaluation when appropriate

The acute event pathway is often a chain rather than a single failure. For example, respiratory infection may thicken secretions, thick secretions may increase suction frequency, repeated suctioning may irritate mucosa and produce bleeding, and bleeding may contribute to clot-related tube obstruction. Similarly, poor fixation may lead to micro-motion, micro-motion may produce granulation tissue or skin injury, and local discomfort may increase agitation and decannulation risk. Recognizing these chains helps clinicians intervene earlier.

Prevention of accidental decannulation requires attention to a set of procedural details that individually appear minor but are collectively important. Ties should be secure but not excessively tight (30). Ventilator tubing should be supported to avoid traction. Children should be stabilized during transfers and procedures. Caregivers and staff should avoid pulling on the tube during suctioning, dressing change, feeding, or repositioning. Agitation, pain, and inadequate sedation during the early postoperative period should be managed because they may increase movement-related risk.

False passage is particularly dangerous because it may create a misleading impression that the tube has been replaced while ventilation remains ineffective. Clues include inability to pass a suction catheter, absent end-tidal carbon dioxide when monitored, poor chest rise, persistent hypoxemia, subcutaneous emphysema, and lack of airflow through the tube. When false passage is suspected, repeated blind attempts should be avoided, and the emergency airway plan should be activated immediately.

Tube obstruction prevention should be individualized. Some children require frequent suctioning because of secretion burden, whereas others may be harmed by unnecessary deep suctioning. Hydration status, fever, airway inflammation, humidifier function, and medication effects can all change secretion viscosity. Families should be taught to recognize changes in baseline patterns, not only absolute thresholds. A child who normally needs suction every few hours but suddenly requires suction every few minutes may be deteriorating even if oxygen saturation remains acceptable.

The evidence base for acute events is uneven. Accidental decannulation is supported by national database analyses, a prospective inpatient cohort, and case-control data (21, 22, 26), whereas catastrophic hemorrhage and false passage are too infrequent for comparative studies and remain characterized mainly by case reports, case series, and expert guidance (16, 20, 23). Evidence for emergency kits, simulation, and standardized rescue algorithms is derived largely from guidelines and quality improvement studies (2, 27–29). These data support consistent preparation and response processes, while the comparative effectiveness of specific preventive bundles and rescue strategies remains uncertain.

Once the early airway threat is controlled, the dominant risk does not disappear; it changes form. The same tube that secures the airway can become a source of chronic irritation, secretion burden, infection uncertainty, skin injury, communication delay, and family workload.

## 5 Long-term respiratory and airway morbidity

Long-term cannulation-related morbidity is the daily reality for many children with tracheostomies. These problems may be less dramatic than acute airway emergencies, but they often determine quality of life, readmission risk, caregiver workload, and decannulation timing. Important domains include peristomal skin injury, local wound complications, chronic secretion burden, respiratory infections, granulation tissue, airway stenosis or malacia, and tube-related discomfort.

Peristomal skin injury is common and often underestimated. The flange and ties exert pressure on delicate pediatric skin, while moisture from secretions and perspiration contributes to maceration. Friction during movement, poor fit, excessive tightness, and short neck anatomy further increase injury risk. Skin findings may include erythema, erosion, ulceration, hypergranulation, drainage, and pain. A systematic review and meta-analysis supports the use of protective dressings, moisture-wicking materials, and skin assessment bundles to reduce tracheostomy-related pressure injury (31).

Peristomal skin problems are not merely cosmetic. Pain and irritation can increase agitation, which may destabilize tube position. Skin breakdown can increase infection risk and make secure fixation difficult. Frequent dressing changes can increase caregiver burden and create variability in tube stabilization. Therefore, skin care should be considered a core safety task. Daily assessment, appropriate dressing selection, moisture control, and individualized fixation are important, particularly for infants, children with heavy secretions, and those with high movement or short necks (2, 31, 32).

Chronic secretion burden is another major problem. Tracheostomy bypasses the upper airway’s normal humidification and filtration functions, leading to drier lower airway secretions. Children with neurologic impairment, aspiration, chronic lung disease, or infection may have increased secretion volume or viscosity. Caregivers may spend substantial time suctioning, cleaning equipment, and responding to alarms. Poor secretion control contributes to tube obstruction, sleep disruption, infection concern, and frequent healthcare contact.

Granulation tissue develops through chronic mechanical irritation and inflammation. It may form at the stoma, along the tract, at the distal tube tip, or within the airway (3, 12, 33, 34). Some lesions are asymptomatic, whereas others cause bleeding, airway narrowing, secretion retention, difficulty with tube changes, or failed decannulation attempts. Management ranges from observation and optimization of tube fit to topical therapy, endoscopic removal, or surgical intervention. The decision should be based on symptoms, location, impact on airway patency, and decannulation plans rather than appearance alone.

Airway stenosis and malacia are particularly important in children with prior prolonged intubation or congenital airway abnormalities (35, 36). Stenosis may result from scarring, mucosal injury, chronic pressure, or repeated instrumentation. Malacia may be primary or secondary and may become more apparent during illness, sleep, or capping trials. These structural problems often determine whether decannulation is possible. They also explain why apparently stable children may fail decannulation despite adequate daytime capping tolerance.

Tube-related morbidity can also affect development and participation. A child with a tracheostomy may have limited mobility because of tubing, suction equipment, and caregiver concern. Feeding and communication may be delayed. Sleep may be interrupted by suctioning, alarms, or caregiver checks. These experiences occur during important developmental periods and may influence family routines, rehabilitation engagement, and psychosocial adaptation. Long-term cannulation should therefore be evaluated not only by medical stability but also by its effect on child development and family function.

Local wound care is a quality indicator because it reflects multiple dimensions of practice: tube fit, dressing choice, tie tension, secretion control, caregiver technique, and skin surveillance. In children with recurrent skin breakdown, the response should not be limited to changing dressings. The team should reassess tube position, flange pressure, neck anatomy, humidity, secretions, infection, and caregiver burden. Persistent skin injury may be a marker of an unstable care system.

Feeding and nutrition interact with tracheostomy outcomes. Children with chronic respiratory disease may have increased energy expenditure, while those with neurologic impairment may have oral motor dysfunction or aspiration risk. Poor nutrition can impair wound healing, immune function, and rehabilitation. Conversely, feeding intolerance may increase reflux and aspiration, contributing to respiratory symptoms. Dietitians and feeding specialists should be included when growth faltering, recurrent infection, or dysphagia is present.

Speech and communication should be addressed early. For preverbal children, tracheostomy may alter vocal exploration and parent-child interaction. For older children, inability to speak may cause frustration, fear, and social withdrawal. Alternative communication strategies, speaking valve assessment, and speech therapy can improve participation. These interventions should not be delayed until decannulation appears imminent, because communication development is time sensitive.

School and social participation are often underdiscussed outcomes. Children with long-term tracheostomies may require specialized school nursing, emergency plans, transportation support, and staff education. Families may avoid school or community settings because of fear of infection or tube emergencies. As children stabilize, care plans should gradually shift from survival to participation, including safe integration into developmentally appropriate environments.

Evidence for chronic cannulation morbidity is dominated by retrospective cohorts, descriptive series, and extrapolation from wound-care or airway literature. Among these domains, the clearest comparative evidence concerns prevention of tracheostomy-related pressure injury (31, 32). Thresholds for endoscopic surveillance, intervention for asymptomatic granulation tissue, and reassessment of tube fit have not been standardized (33–36). Decisions should therefore be guided by symptoms, airway function, growth, and decannulation plans, until prospective studies establish surveillance intervals and patient-centered outcomes.

## 6 Respiratory infection, readmission, and caregiver burden

Respiratory infection is one of the most important drivers of long-term morbidity in children with tracheostomies. It is also one of the hardest domains to define consistently. Many children with chronic tracheostomies have colonized airways, and positive tracheal aspirate cultures are common. Colonization, increased secretions, viral illness, aspiration, tracheitis, and pneumonia may overlap clinically. This creates uncertainty about when to culture, when to treat, and how to avoid unnecessary antibiotic exposure (37–42).

The distinction between colonization and infection is clinically essential. A culture result without compatible clinical deterioration may represent colonization rather than disease. Conversely, a child with fever, increased work of breathing, rising oxygen requirement, worsening secretions, and new radiographic change may require treatment even if cultures are difficult to interpret. The ATS guideline recommends tracheal aspirate cultures during acute respiratory exacerbations to support treatment decisions, while discouraging routine surveillance cultures as a monitoring strategy (1). This approach aligns with antimicrobial stewardship and avoids overinterpretation of chronic colonization.

Antimicrobial management varies substantially across centers because diagnostic criteria are inconsistent and pediatric comparative evidence is limited (38, 40). Treatment decisions should integrate clinical deterioration, illness severity, changes in respiratory support and secretions, prior microbiology and susceptibility patterns, and response to therapy. Inhaled antibiotics may be considered for selected lower-airway infections, whereas prophylactic systemic antibiotics require a separate indication; evidence remains insufficient to standardize either approach or treatment duration.

Respiratory infections are major drivers of healthcare utilization. Children with existing tracheostomies experience frequent hospitalizations, with respiratory diagnoses representing a major proportion of admissions (17, 43). Emergency department visits and rehospitalizations are also common after tracheostomy placement, especially in medically complex children (18, 19). These events are not only markers of disease severity; they also reflect the stability of home care, caregiver confidence, outpatient accessibility, and the clarity of escalation plans.

Caregiver burden is tightly linked to infection and readmission. Families often live with constant vigilance: monitoring secretions, suction frequency, oxygen saturation, tube position, fever, and alarms. Recurrent respiratory symptoms may produce a cycle of anxiety, emergency visits, treatment, temporary improvement, and recurrence. Over time, this cycle can erode caregiver confidence, sleep, employment, and social participation (44–46). In some families, the psychological burden of preventing tube-related emergencies is as significant as the medical burden itself.

Evaluation of recurrent respiratory symptoms should also include swallowing safety, aspiration risk, humidification, secretion clearance, tube fit, vaccination status, nutrition, caregiver education, and access to timely outpatient advice. Recurrent presentations labeled as tracheitis may reflect aspiration, inadequate humidification, secretion retention, or uncertainty about warning signs. Identifying these contributing factors may reduce repeated emergency visits and antibiotic exposure.

Future studies should aim to standardize definitions for pediatric tracheostomy-associated respiratory infection. Clinically useful definitions should combine symptoms, changes in respiratory support, secretion characteristics, systemic inflammatory signs, microbiology, and radiographic findings when available. They should also distinguish between outpatient-managed exacerbations, emergency visits, hospitalizations, and ICU-level deterioration. Without shared definitions, evidence-based antimicrobial strategies and quality improvement benchmarks will remain difficult to develop (47).

Readmission should be interpreted as a clinically meaningful outcome, but not all readmissions are preventable. Some reflect unavoidable disease severity. Others may reflect modifiable gaps such as unclear discharge instructions, equipment problems, delayed outpatient access, inadequate caregiver confidence, or unresolved swallowing and secretion issues. Quality improvement efforts should therefore examine the reason for each readmission, the timeline after discharge, and whether earlier intervention could have changed the course.

Caregiver burden should be measured rather than assumed. Parents may become highly skilled but still experience exhaustion, hypervigilance, financial strain, social isolation, and sleep deprivation. Some families may hesitate to report difficulty because they fear delaying discharge or being judged as incapable. Clinicians should ask directly about stress, backup support, night coverage, and confidence in emergency response. Supporting caregivers is a safety intervention, not only a psychosocial service.

Evidence for tracheostomy-associated respiratory infection remains low certainty because case definitions are heterogeneous, most studies are retrospective, and comparative evidence on antimicrobial selection, route, and duration is limited (37, 40). Caregiver burden is consistently reported across qualitative studies and meta-analysis, although effective support interventions have not been established (44–46). Research priorities include consensus infection definitions, prospective evaluation of antimicrobial strategies, and comparative studies of caregiver-support interventions.

## 7 Standardized inpatient and multidisciplinary care

Inpatient standardization is one of the most practical ways to reduce preventable post-tracheostomy adverse events. Standardization ensures reliable completion of essential safety steps while allowing individualized decisions within a clear framework. Key elements include appropriate tube selection, secure fixation, humidification, suctioning, skin care, tube change planning, emergency equipment availability, staff education, and structured communication at handoffs (2). Table 3 summarizes core inpatient management elements.

**Table 3:** Key elements of standardized inpatient management after pediatric tracheostomy.

Domain	Core elements	Pediatric rationale	Evidence basis
Tube selection and fit	Size, length, curvature, cuff need, ventilator requirement, growth reassessment	Poor fit contributes to leakage, pressure injury, distal trauma, granulation tissue, tube displacement, or inadequate ventilation	Guideline/expert consensus (1, 2)
Fixation	Standard tie technique, tension checks, tubing support, securement during transfers	Reduces accidental decannulation and pressure-related skin injury	Guideline/expert consensus (2, 30)
Humidification	Individualized active or passive humidification according to secretions and support mode	Prevents thick secretions and tube obstruction after bypassing upper airway humidification	Guideline/expert consensus (1, 2)
Suctioning	Clinical indication-based suctioning, appropriate catheter size and depth, trauma avoidance	Maintains patency while limiting mucosal injury and bleeding	Guideline/expert consensus (1, 2)
Skin and stoma care	Daily inspection, cleansing, moisture management, protective dressing	Prevents pressure injury, infection, pain, and loss of secure tube fixation	Systematic review/guideline (31, 32)
Tube change protocol	Experienced personnel, backup equipment, timing based on tract maturity and clinical need	Reduces false passage and emergency complications during tube replacement	Guideline/expert consensus (2, 24)
Emergency preparedness	Bedside kit, same-size and smaller tube, bag-valve device, oxygen, suction, written plan	Supports rapid response to obstruction, displacement, and inability to ventilate	Guideline/expert consensus (2, 20, 27)
Education and simulation	Staff and caregiver training, crisis rehearsal, competency assessment	Improves response to low-frequency high-risk events	Observational/QI studies (28, 29, 48)
Multidisciplinary coordination	PICU, otolaryngology, pulmonology, nursing, respiratory therapy, speech, nutrition, rehabilitation, social work	Aligns airway care, discharge readiness, function, and family support	Observational/QI studies (49, 50)

Tube selection should consider age, airway anatomy, indication for tracheostomy, ventilator requirements, aspiration risk, tube length, curvature, and the need for a cuff. Cuffed tubes may be necessary for positive-pressure ventilation or aspiration management, but cuff pressure and mucosal injury risk require attention (2). A poorly matched tube can contribute to leakage, pressure injury, distal trauma, granulation tissue, or inadequate ventilation. The correct tube is therefore a dynamic choice that may change as the child grows or clinical needs evolve.

Humidification and suctioning are central to safety. Because tracheostomy bypasses the upper airway, inspired gas is less humidified and filtered. Inadequate humidification can lead to thick secretions and tube obstruction. Overly aggressive suctioning can cause mucosal trauma, bleeding, discomfort, and vagal responses. A balanced approach uses clinical signs—such as secretion burden, breath sounds, work of breathing, oxygenation, ventilator alarms, and suction resistance—to guide interventions rather than relying only on fixed schedules (2, 5, 6).

Tube changes require planning. Early tube change should be performed by experienced personnel with appropriate support, especially before tract maturation (2, 24, 32). Routine changes after stabilization should be performed according to institutional protocol, manufacturer guidance, tube condition, and clinical need. Families should eventually learn tube change or emergency replacement procedures when appropriate, but competency should be demonstrated before discharge rather than assumed.

Emergency preparedness is essential because catastrophic events are usually time sensitive. Bedside emergency supplies should be complete, visible, and standardized. Written emergency algorithms should be clear and specific to the child when needed. Staff caring for tracheostomized children outside the ICU should receive recurrent training, because low-frequency high-risk events are difficult to manage without practice. Simulation can improve team coordination, clarify roles, and reveal equipment or communication gaps before a real crisis (28, 29, 48).

Multidisciplinary tracheostomy teams are increasingly used as an organizational model for coordinating care. These teams may include pediatric intensivists, otolaryngologists, pulmonologists, respiratory therapists, nurses, speech and language therapists, dietitians, rehabilitation clinicians, social workers, and discharge coordinators. Observational studies suggest that multidisciplinary approaches may reduce adverse events, improve education, and decrease emergency department utilization (49–51). Their value lies not only in expertise but also in shared ownership of the child's full care pathway.

The need for multidisciplinary care is particularly clear when assessing discharge readiness. A child may be stable from an airway perspective but not ready for home if caregivers cannot perform suctioning, equipment is unavailable, swallowing risk is unaddressed, or follow-up is unclear. Conversely, a child may remain hospitalized longer than necessary if there is no coordinated pathway for training and equipment approval. Multidisciplinary programs can reduce these mismatches by aligning clinical stability, caregiver competence, and system readiness.

Standardized order sets and checklists can improve reliability. Examples include postoperative tracheostomy care bundles, first tube change checklists, emergency kit checklists, skin assessment tools, suction depth documentation, and discharge readiness checklists. These tools are most effective when embedded into routine workflow rather than maintained as separate documents.

Education must extend beyond specialized units (52, 53). Children with tracheostomies may be cared for in emergency departments, imaging suites, operating rooms, inpatient wards, rehabilitation units, schools, and transport settings. Staff in these locations may be less familiar with pediatric tracheostomy emergencies. Targeted education for non-PICU environments can reduce risk during procedures, imaging, transport, and unexpected deterioration.

Across these inpatient elements, the strength of evidence is heterogeneous. Tube selection, humidification, suctioning, skin care, emergency equipment, and early tube-change planning are supported mainly by pediatric guidelines and expert consensus. Multidisciplinary teams, simulation, and structured checklists rely largely on observational and quality improvement studies (28, 29, 48–50). Implementation should therefore be accompanied by prospective measurement of accidental decannulation, tube obstruction, pressure injury, unplanned ICU transfer, readmission, and caregiver competency, allowing local benefit and resource requirements to be assessed.

## 8 Hospital-to-home transition and airway safety

**Figure 3:**
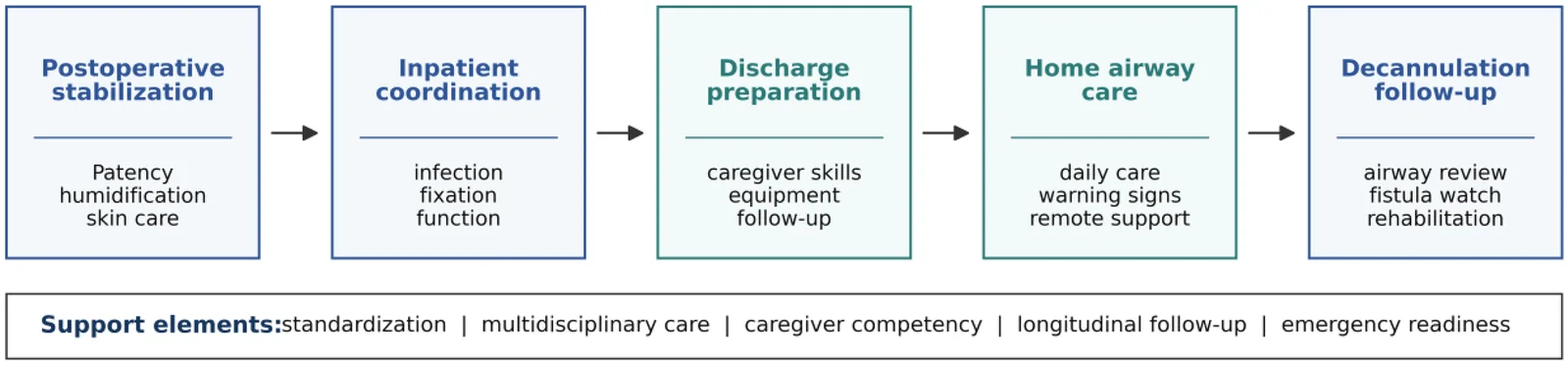
Implementation pathway for longitudinal pediatric tracheostomy care from postoperative stabilization to home care and decannulation follow-up.

Hospital-to-home transition is one of the most vulnerable phases in pediatric tracheostomy care. In hospital, adverse events are recognized by trained staff in monitored environments; at home, families become the primary observers and first responders. This transfer of responsibility requires technical competence, emotional readiness, reliable equipment, and defined access to urgent support (1, 10, 44, 45, 54–56). Figure 3 translates the longitudinal framework into an implementation pathway spanning postoperative stabilization, inpatient coordination, discharge preparation, home airway care, and decannulation follow-up.

Transition planning should begin soon after tracheostomy placement. Early initiation provides time for families to understand long-term care requirements, observe and practice care, and develop confidence. It also allows the team to identify language needs, limited caregiver availability, transportation barriers, and home-equipment concerns before discharge.

Caregiver training should require demonstrated competence in routine tracheostomy care and emergency response. Families need repeated supervised practice in suctioning, humidification, skin and tie care, equipment troubleshooting, recognition of tube obstruction, and response to accidental decannulation. Competency assessment should also include simulated scenarios involving ventilator disconnection, bleeding, oxygen desaturation, and equipment failure (28, 29). Table 4 summarizes minimum discharge-readiness expectations and high-risk signals requiring additional planning.

**Table 4:** Discharge readiness and caregiver capability assessment for children with tracheostomies.

Readiness domain	Minimum expectation before discharge	High-risk signal requiring additional planning
Routine tracheostomy care	Caregiver demonstrates cleaning, dressing, tie checks, and routine observation	Caregiver can describe steps but cannot perform them reliably
Suctioning and secretion assessment	Caregiver recognizes when suction is needed and performs it safely	Caregiver suctions only by schedule or cannot interpret secretion changes
Tube obstruction response	Caregiver recognizes early obstruction signs and initiates emergency steps	Caregiver waits for severe desaturation or does not know first actions
Accidental decannulation response	Caregiver understands child-specific airway plan and replacement/escalation steps	Caregiver cannot complete key emergency steps during simulation or cannot access the backup tube promptly
Equipment use	Caregiver operates suction, humidification, pulse oximeter, oxygen, and ventilator when applicable	Caregiver cannot troubleshoot alarms, power failure, or equipment malfunction
Nighttime supervision	Reliable plan for awake trained caregiver or nursing support when required	No backup caregiver, caregiver exhaustion, or unclear night coverage
Emergency pathway	Family knows when and how to contact emergency services and specialty team	Unclear escalation plan, long travel distance, or poor access to emergency care
Home environment and supplies	Adequate space, electricity, hygiene, replacement tubes, suction supplies, transport plan	Supply insecurity, unstable electricity, transportation barriers, or unsafe storage
Follow-up plan	Early outpatient or telehealth follow-up scheduled with clear responsibilities	No defined follow-up, fragmented specialty care, or poor communication channels
Caregiver burden and resilience	Family has sufficient support and realistic understanding of care demands	High anxiety, language barriers, financial stress, or single unsupported caregiver

Training should establish the child's baseline care needs, clinically important changes, and the appropriate level of response. Changes in secretion characteristics, suction frequency, work of breathing, oxygen saturation, ventilator alarms, tube position, temperature, bleeding, or skin integrity should have predefined action plans. Caregivers should be able to distinguish concerns suitable for home management from those requiring same-day clinical advice or emergency services.

Nighttime care deserves special attention. Many high-risk events occur when supervision is reduced. Children who are ventilator dependent, have unstable airways, require frequent suctioning, or have limited physiologic reserve may need continuous awake observation by a trained caregiver or nurse (1, 10). Families may underestimate the burden of night care until after discharge. Before discharge, the team should explicitly assess who will provide nighttime supervision, whether backup caregivers exist, and how fatigue will be managed.

Home equipment readiness is another determinant of safety. Essential equipment may include suction devices, humidification systems, oxygen delivery devices, pulse oximetry, ventilator support when needed, backup power, replacement tubes, emergency supplies, and transport equipment (10). Families should be able to maintain equipment, respond to malfunction, and obtain replacement supplies promptly.

The social context of home care cannot be ignored. Housing conditions, electricity reliability, transportation, caregiver literacy, language barriers, financial stress, distance from hospital, and availability of home nursing all influence safety. A technically competent family may still be at risk if there is no backup caregiver, unreliable transportation, or inadequate access to supplies. Discharge planning should therefore include social work support, caregiver-burden assessment, and a feasible emergency plan.

Early follow-up should address problems that emerge after families begin independent care at home. Outpatient review, telephone access, telehealth, home-nursing feedback, and clear escalation pathways may reduce avoidable emergency visits. Assessment should include tube position, skin condition, secretion burden, respiratory symptoms, feeding, sleep, caregiver confidence, and equipment function.

Discharge readiness requires multidisciplinary agreement on tract and airway stability, respiratory-support needs, equipment settings, daily care competence, swallowing and communication, nutrition, home resources, and the overall risk plan. Responsibilities should be assigned explicitly before discharge to prevent gaps between clinical, technical, functional, and family preparation.

Telehealth can support families after discharge, particularly for early skin concerns, equipment questions, secretion changes, and caregiver uncertainty. It cannot replace emergency evaluation for acute deterioration, but it may reduce avoidable visits and improve confidence. Programs should define which concerns are appropriate for telehealth and which require immediate in-person assessment.

Guidelines, qualitative studies, and practice surveys support caregiver training, awake supervision for selected high-risk children, equipment readiness, and early follow-up (1, 10, 44, 45, 54–56). Comparative evidence linking individual transition components to readmission, emergency events, or mortality remains limited, and program outcomes are influenced by disease severity, home-nursing availability, and social resources. Future studies should establish competency thresholds, evaluate alternatives where home nursing is unavailable, and measure emergency events, caregiver burden, and sustained home-care stability.

## 9 Decannulation and functional outcomes

Decannulation is a major goal for many children, but it should be approached as a staged clinical decision rather than a simple procedure. The central question is whether the child can maintain airway patency, secretion clearance, respiratory stability, and swallowing safety without the tracheostomy. These abilities may improve over time as lung disease stabilizes, airway reconstruction is completed, neurologic status evolves, or the child grows. However, some children remain permanently dependent on tracheostomy because of airway, respiratory, or neurologic limitations.

Airway evaluation is central to decannulation planning. Flexible or rigid endoscopy may identify suprastomal collapse, granulation tissue, subglottic stenosis, tracheomalacia, vocal cord immobility, or other lesions that could prevent safe tube removal (1, 57, 58). Structural findings must be interpreted in context. Mild narrowing may be clinically acceptable in one child but problematic in another with poor reserve, heavy secretions, or sleep-related hypoventilation. Endoscopy is therefore necessary but not always sufficient.

Progressive capping or downsizing is commonly used to assess tolerance. These strategies can test airflow through the upper airway and the child's ability to manage secretions. However, daytime capping tolerance does not guarantee nighttime safety. Sleep may reveal hypoventilation, dynamic obstruction, or oxygen desaturation that is not evident when awake. Polysomnography or overnight monitored assessment may be useful in selected children, particularly those with neurologic impairment, airway malacia, obesity, craniofacial abnormalities, or suspected sleep-disordered breathing (1, 57).

Swallowing and aspiration risk should be considered before and after decannulation. Children with tracheostomies may have dysphagia related to neurologic disease, altered airway sensation, prolonged critical illness, reduced laryngeal elevation, or chronic aspiration (59–61). Persistent aspiration can drive respiratory infections and delay decannulation even when airway anatomy appears adequate. Speech and language therapists, occupational therapists, dietitians, and rehabilitation teams are therefore important contributors to decannulation planning.

Communication and phonation are also central outcomes. Tracheostomy may limit voice production, reduce participation, and affect development. Speaking valves may support airflow through the upper airway and facilitate communication in selected children, but their use requires careful assessment of airway patency, secretion burden, respiratory support needs, and tolerance (59, 60). Communication goals should be individualized and introduced early when feasible rather than deferred until after decannulation.

Persistent tracheocutaneous fistula is a common post-decannulation issue, particularly after prolonged cannulation (62–64). Some fistulas close spontaneously, whereas others require surgical closure. Persistent fistula may cause air leak, skin irritation, voice changes, cosmetic concern, and family dissatisfaction. For families, decannulation success includes not only tube removal but also healing, voice, comfort, and return to normal activities. Follow-up after decannulation should therefore continue until functional recovery is clear.

Long-term outcomes should extend beyond survival and decannulation status. Important pediatric outcomes include feeding, growth, sleep, communication, mobility, school participation, neurodevelopment, family quality of life, and caregiver mental health. These outcomes are inconsistently measured in existing studies. As a result, clinicians may know whether a child survived or was decannulated but not whether the child regained meaningful function or whether the family's burden improved. Future research should incorporate child- and family-centered outcomes alongside airway and hospitalization metrics.

Decannulation readiness is dynamic. Intercurrent viral infection or aspiration may temporarily reduce readiness, while growth-related changes in airway caliber or tube fit may alter capping tolerance before decannulation. Conversely, readiness may improve after airway surgery, lung growth, better nutrition, or improved neurologic control. Regular reassessment prevents both premature decannulation and unnecessary prolonged cannulation.

The risks of delayed decannulation should also be acknowledged. Prolonged cannulation may increase exposure to infection, skin injury, granulation tissue, communication limitations, caregiver burden, and persistent tracheocutaneous fistula (62). While safety must remain paramount, indefinite deferral without reassessment may impose avoidable burdens. A structured decannulation pathway can help balance caution with progress.

Post-decannulation surveillance should include respiratory and functional review. Families should be advised about signs of airway obstruction, persistent fistula, voice change, exertional limitation, sleep symptoms, and recurrent infection. For some children, removal of the tube reveals previously masked upper airway or sleep-related problems. Continued access to airway specialists after decannulation is therefore important.

Functional recovery should be individualized. Some children may focus on speech, others on feeding, school attendance, physical activity, or caregiver independence. Rehabilitation goals should be realistic and developmentally appropriate. The best outcome is not simply absence of a tracheostomy tube, but the child’s ability to participate as fully as possible within the limits of underlying disease.

Decannulation evidence illustrates the tension between standardization and individualization. A systematic review identified substantial variation in the use of endoscopy, capping or downsizing, polysomnography, and post-decannulation observation (57). Most protocols derive from single-center observational cohorts, and validated thresholds for sleep gas exchange, secretion burden, cough effectiveness, or swallowing safety are lacking. Endoscopic airway assessment is widely incorporated, while the incremental value of routine polysomnography over selective overnight monitoring remains unresolved. Comparative multicenter studies are needed to distinguish protocol components associated with successful decannulation from those that primarily reflect local resources and risk tolerance.

## 10 Discussion

### 10.1 From complications to airway-safety pathways

The central contribution of the framework is its organizing logic. Early-versus-late categories describe timing but do not show how risks change across settings or how one problem may alter the probability of another. A phase-based pathway links changing risk to surveillance and escalation before overt deterioration.

Existing guidance provides the component practices of safe pediatric tracheostomy care (1–5). The framework reorganizes them by phase, care setting, responsibility, escalation trigger, and outcome. Table 1 shows this distinction, while Figures 1–3 translate the model into risk mapping, action-oriented triage, and transition implementation.

### 10.2 Integrating care across settings

The operational value of the framework lies at transitions between care settings. Planning should begin at tracheostomy placement and connect early airway risk, caregiver competency, home respiratory support, and follow-up responsibilities before each transfer.

Decannulation should remain part of this longitudinal plan, with reassessment based on airway patency, respiratory stability, secretion clearance, swallowing, sleep, and the underlying disease trajectory. This approach limits both premature decannulation and avoidable prolonged cannulation.

Table 5 operationalizes the pathway by specifying the dominant risk, required integration, and escalation trigger for each care phase. It is intended for local adaptation according to patient characteristics and available resources.

**Table 5:** Implementation-oriented longitudinal airway-safety pathway for children with tracheostomies.

Care phase	Main risk logic	Required integration	Escalation trigger
PICU decision and early postoperative period	Airway stabilization reduces intubation-related risk but creates new risks of bleeding, false passage, air leak, obstruction, and accidental decannulation	Airway plan, tube size, rescue route, first tube change, sedation and analgesia, emergency equipment, and team briefing.	Bleeding, inability to pass suction catheter, unexplained desaturation, loss of exhaled volume, subcutaneous emphysema, or difficult reinsertion.
Inpatient stabilization	Risk shifts from immediate airway loss to secretion control, skin integrity, infection interpretation, tube fit, and staff reliability outside the ICU.	Standardized humidification, suction depth, stoma care, fixation, ventilator or oxygen settings, handoff content, and caregiver teaching.	Increasing suction frequency, recurrent alarms, stomal breakdown, fever with respiratory decline, repeated accidental decannulation or tube displacement, or staff uncertainty about rescue steps.
Discharge preparation	Clinical stability is insufficient if caregiver skills, equipment, night supervision, supply continuity, and escalation plans are unreliable.	Competency-based training, consistent supervised demonstration of routine tracheostomy care, simulation, home equipment, backup caregivers, transportation, early follow-up, and written emergency plan.	Failed simulation, no trained backup caregiver, unsafe night plan, supply insecurity, unreliable power, or no defined outpatient contact.
Home care and recurrent illness	Home events reflect both biology and system reliability: infection, aspiration, humidification, equipment, caregiver fatigue, and access to advice interact.	Early outpatient review, telehealth boundaries, home nursing feedback, antimicrobial stewardship, aspiration assessment, vaccination, nutrition, and caregiver support.	Rising oxygen or ventilator need, fever plus increased work of breathing, bloody secretions, frequent emergency visits, equipment failure, or caregiver exhaustion.
Decannulation and functional recovery	Tube removal is safe only when airway patency, ventilation and oxygenation during sleep, secretion clearance, swallowing safety, and family readiness are aligned.	Endoscopy, capping or downsizing, selective overnight monitoring or polysomnography, swallowing and speech evaluation, rehabilitation goals, and post-decannulation surveillance.	Failed capping, stridor, sleep desaturation, recurrent aspiration, poor secretion clearance, persistent fistula, voice change, or recurrent respiratory admissions.

### 10.3 Evidence gaps and research priorities

Progress is limited by inconsistent definitions, particularly for respiratory infection, tube obstruction, accidental decannulation, decannulation failure, and caregiver burden. Most implementation strategies, including simulation, emergency kits, caregiver checklists, multidisciplinary clinics, and telehealth, are supported by observational or quality improvement data, with limited evidence on their comparative effectiveness, optimal combination, and sustainability—questions that require implementation research examining how such strategies are adopted, delivered, and sustained in routine practice.

The framework has limitations that reflect its scientific basis. Its components are supported by evidence of unequal strength, ranging from clinical guidelines and systematic reviews to retrospective cohorts, qualitative studies, and quality improvement reports. The phase-to-phase links, escalation thresholds, and allocation of professional responsibility have not been evaluated as a complete pathway. Development did not include formal consensus methods, stakeholder co-design, or prospective validation. Applicability may therefore vary with case mix, staffing, home-nursing availability, equipment supply, and access to specialist follow-up.

Future work should prioritize pediatric-specific outcome frameworks. Relevant outcomes should include acute airway events, infection-related healthcare utilization, home care stability, caregiver confidence, sleep disruption, swallowing, phonation, school participation, decannulation, persistent fistula, and child- and family-reported quality of life. Multicenter registries would be particularly useful because individual centers often have limited sample size and heterogeneous case mix. Such registries should include clinical, airway, respiratory support, social, and functional variables.

Validation should proceed in stages. Content validity should first be assessed by multidisciplinary clinicians and family caregivers, followed by testing of the feasibility and inter-rater reliability of phase-specific variables and escalation triggers. Prospective multicenter studies should then evaluate whether pathway implementation improves the outcomes listed above without delaying discharge or creating disproportionate resource use. External evaluation across health systems with different home-care resources is required before broad implementation.

Practice variation is greatest for tracheostomy timing, cuffed tube use, antimicrobial strategy, and the intensity of decannulation testing. As outlined above, decisions in each area should be individualized within standardized care pathways according to diagnosis, airway anatomy, ventilatory needs, aspiration risk, and sleep-related symptoms; each area is also a priority for comparative study (1, 40).

Implementation must be resource-sensitive. Where home nursing, equipment supply, or specialist access is limited, minimum safety requirements should include a written emergency plan, reliable suction and backup tube access, competency-confirmed caregiver training, a defined escalation contact, and early post-discharge review (51). These constraints are directly relevant to settings such as China and should be treated as determinants of safety and equity.

Table 6 summarizes the evidence base, qualitative confidence, major limitations, and research priorities across the principal care domains.

**Table 6:** Qualitative appraisal of evidence supporting major pediatric tracheostomy care domains.

Care domain	Principal evidence base	Qualitative confidence	Main limitations and research priority
Emergency airway planning and tube rescue	Guidelines, expert consensus, observational studies, and quality improvement reports (1, 2, 20–22, 27–29)	Moderate	Clinical rationale and recommendations are consistent, but the comparative effectiveness of rescue algorithms and prevention bundles remains uncertain.
Humidification, suctioning, tube fit, and routine tube care	Guidelines and foundational reviews (1, 2, 5, 6)	Moderate	Direct pediatric comparative studies are scarce, and optimal protocols vary with tube characteristics, secretion burden, and respiratory support.
Peristomal skin and pressure-injury prevention	Systematic review and meta-analysis, guideline, and evidence review (2, 31, 32)	Moderate	Intervention definitions and care bundles vary; pediatric subgroup data and comparative dressing studies remain limited.
Caregiver competency, simulation, and discharge readiness	Guidelines, scoping and qualitative studies, and quality improvement reports (1, 28, 29, 44, 45, 52–56)	Low to moderate	Competency thresholds and their associations with emergency events, readmission, and sustained home stability require prospective evaluation.
Multidisciplinary care and standardized pathways	Observational cohorts and quality improvement studies (49–53)	Low to moderate	Selection bias, center effects, and bundled interventions limit attribution of benefit to individual components.
Tracheostomy-associated respiratory infection management	Guideline, scoping and systematic reviews, and multicenter observational studies (1, 37–42)	Low	Case definitions are inconsistent, and evidence remains limited for antimicrobial selection, route, duration, prophylaxis, and inhaled therapy.
Decannulation assessment and follow-up	Guideline, systematic review, and single-center cohorts (1, 57, 58, 62–64)	Low to moderate	Protocols vary in endoscopy, capping, sleep testing, and observation; comparative studies and multicenter validation are limited.
Swallowing, communication, and family-centered outcomes	Observational and qualitative studies, systematic review, and meta-analysis (44–46, 59–61)	Low	Outcome measures are heterogeneous, and intervention studies linking functional support to long-term child and family outcomes are scarce.

Confidence ratings are qualitative author appraisals based on study design, pediatric directness, consistency, and clinical applicability. Formal GRADE methods were not applied.

### 10.4 Implications for pediatric pulmonology

Pediatric pulmonology is positioned to coordinate the respiratory domains that persist across care settings, including airway clearance, aspiration, ventilatory support, sleep-related hypoventilation, hypoxemia, upper-airway obstruction, infection patterns, and decannulation readiness. Its role is integrative, linking these domains with otolaryngology, intensive care, rehabilitation, nursing, and family caregivers.

Implementation priorities are a defined airway rescue plan, standardized ward management, competency-confirmed caregiver training, reliable equipment and escalation access, early post-discharge review, and scheduled reassessment of function and decannulation potential. Programs should prospectively measure safety events, healthcare use, caregiver outcomes, and resource burden rather than infer benefit from pathway adoption alone.

## 11 Conclusions

Post-tracheostomy adverse events in children form a continuum of airway-safety risks across care settings. Acute events such as hemorrhage, accidental decannulation, false passage, air leak syndromes, and tube obstruction remain central threats because they can rapidly cause ventilatory failure. Long-term respiratory, airway, functional, and family consequences shape daily life and recovery through their effects on respiratory stability, healthcare use, communication, feeding, and caregiver workload.

Clinical priorities should be defined by which child is at risk, at which phase of care, and which modifiable care process requires intervention. This assessment integrates airway anatomy, respiratory physiology, secretion management, infection surveillance, sleep-related assessment, caregiver capability, home resources, and functional goals.

Optimal care combines safe surgical placement with standardized inpatient management, reliable emergency preparedness, competency-based caregiver training, a supported hospital-to-home transition, structured follow-up, and planned decannulation assessment. The goal is to reduce preventable events, support functional recovery, and improve child and family quality of life throughout tracheostomy dependence. A measurable pathway should document each child's airway rescue plan, secretion and infection strategy, caregiver competency, home support, follow-up responsibilities, and periodically reassessed decannulation plan.

## References

[1] Amin R Agarwal A Chiang J Collaco JM Cristea AI Propst EL. Care of infants and children with tracheostomies: an official American Thoracic Society clinical practice guideline. Am J Respir Crit Care Med. (2025) 211:2001–20. 10.1164/rccm.202508-2055ST 41123183 PMC12618984

[2] Volsko TA Parker SW Deakins K Walsh BK Fedor KL Valika T. AARC Clinical practice guideline: management of pediatric patients with tracheostomy in the acute care setting. Respir Care. (2021) 66:144–55. 10.4187/respcare.08137 33380501

[3] Dal'Astra APL Quirino AV Caixeta JAS Avelino MAG. Tracheostomy in childhood: review of the literature on complications and mortality over the last three decades. Braz J Otorhinolaryngol. (2017) 83:207–14. 10.1016/j.bjorl.2016.04.005 27256033 PMC9442684

[4] Vargas M Servillo G. Tracheostomy in children: a narrative review. Front Surg. (2026) 13:1787750. 10.3389/fsurg.2026.1787750 41993789 PMC13079020

[5] Fuller C Wineland AM Richter GT. Update on pediatric tracheostomy: indications, technique, education, and decannulation. Curr Otorhinolaryngol Rep. (2021) 9:188–99. 10.1007/s40136-021-00340-y 33875932 PMC8047564

[6] Watters KF. Tracheostomy in infants and children. Respir Care. (2017) 62:799–825. 10.4187/respcare.05366 28546379

[7] Willis LD. Pediatric tracheostomy year in review. Respir Care. (2024) 69:1025–32. 10.4187/respcare.11932 38626953 PMC11298220

[8] Roberts J Powell J Begbie J Siou G McLarnon C Welch A. Pediatric tracheostomy: a large single-center experience. Laryngoscope. (2020) 130:E375–80. 10.1002/lary.28160 31251404

[9] Gergin O Adil EA Kawai K Watters K Moritz E Rahbar R. Indications of pediatric tracheostomy over the last 30 years: has anything changed? Int J Pediatr Otorhinolaryngol. (2016) 87:144–7. 10.1016/j.ijporl.2016.06.018 27368463

[10] Sterni LM Collaco JM Baker CD Carroll JL Sharma GD Brozek JL. An official American Thoracic Society clinical practice guideline: pediatric chronic home invasive ventilation. Am J Respir Crit Care Med. (2016) 193:e16–35. 10.1164/rccm.201602-0276ST 27082538 PMC5439679

[11] Pavone M Verrillo E Onofri A Caggiano S Chiarini Testa MB Cutrera R. Characteristics and outcomes in children on long-term mechanical ventilation: the experience of a pediatric tertiary center in Rome. Ital J Pediatr. (2020) 46:12. 10.1186/s13052-020-0778-8 32005269 PMC6995086

[12] Kremer B Botos-Kremer AI Eckel HE Schlöndorff G. Indications, complications, and surgical techniques for pediatric tracheostomies: an update. J Pediatr Surg. (2002) 37:1556–62. 10.1053/jpsu.2002.36184 12407539

[13] Mahida JB Asti L Boss EF Shah RK Deans KJ Minneci PC. Tracheostomy placement in children younger than 2 years: 30-day outcomes using the National Surgical Quality Improvement Program Pediatric. JAMA Otolaryngol Head Neck Surg. (2016) 142:241–6. 10.1001/jamaoto.2015.3302 26822902

[14] Douglas CM Poole-Cowley J Morrissey S Kubba H Clement WA Wynne DM. Paediatric tracheostomy: an 11-year experience at a Scottish paediatric tertiary referral centre. Int J Pediatr Otorhinolaryngol. (2015) 79:1673–6. 10.1016/j.ijporl.2015.07.022 26255606

[15] Ishaque S Haque A Qazi SH Mallick H Nasir S. Elective tracheostomy in critically ill children: a 10-year single-center experience from a lower-middle income country. Cureus. (2020) 12:e9080. 10.7759/cureus.9080 32789032 PMC7416984

[16] Pérez-Ruiz E Caro P Pérez-Frías J Cols M Barrio I Torrent A. Paediatric patients with a tracheostomy: a multicentre epidemiological study. Eur Respir J. (2012) 40:1502–7. 10.1183/09031936.00164611 22496314

[17] Zhu H Das P Roberson DW Jang J Skinner ML Paine M. Hospitalizations in children with preexisting tracheostomy: a national perspective. Laryngoscope. (2015) 125:462–8. 10.1002/lary.24797 24986601

[18] Beams DR Chorney SR Kou YF Teplitzky TB Wynings EM Johnson RF. Emergency department visits and hospitalizations after pediatric tracheostomy. Laryngoscope. (2023) 133:2018–24. 10.1002/lary.30416 36177909

[19] Tarfa RA Morris J Melder KL McCoy JL Tobey ABJ. Readmissions and mortality in pediatric tracheostomy patients: are we doing enough? Int J Pediatr Otorhinolaryngol. (2021) 145:110704. 10.1016/j.ijporl.2021.110704 33882340

[20] Bontempo LJ Manning SL. Tracheostomy emergencies. Emerg Med Clin North Am. (2019) 37:109–19. 10.1016/j.emc.2018.09.010 30454773

[21] Heninger J Ghosh A Rowland M Hazkani I Valika T Cheon EC. Accidental tracheostomy decannulation: risk factors and complications in pediatric patients using the NSQIP-P database. Int J Pediatr Otorhinolaryngol. (2024) 187:112174. 10.1016/j.ijporl.2024.112174 39622094

[22] Wynings EM Breslin N Brooks RL Brown AF Bailey CH Whitney C. Accidental tracheostomy decannulations in children: a prospective cohort study of inpatients. Laryngoscope. (2023) 133:963–9. 10.1002/lary.30250 35712851

[23] Stramiello JA Friesen TL Rao A Ratnayaka K Moore J El-Said H. Aortopulmonary collaterals: an etiology for pediatric tracheostomy hemorrhage. Int J Pediatr Otorhinolaryngol. (2022) 158:111123. 10.1016/j.ijporl.2022.111123 35483154

[24] Gumussoy M. Pediatric tracheotomy: comparison of surgical technique with early and late complications in 273 cases. Pak J Med Sci. (2019) 35:247–51. 10.12669/pjms.35.1.132 30881432 PMC6408647

[25] Song J-J Choi IJ Chang H Kim DW Chang HW Park G-H. Pediatric tracheostomy revisited: a nine-year experience using horizontal intercartilaginous incision. Laryngoscope. (2015) 125:485–92. 10.1002/lary.24882 25130887

[26] Villarroel GS Faúndez M Jalil YF Oyarzún IJ Fernandez TR Barañao PI. Factors associated with accidental decannulation in tracheostomized children. Respir Care. (2023) 68:173–9. 10.4187/respcare.09673 37610360 PMC9994284

[27] Cherches A Wang A Patterson RH Lee J Cheng J. Preventing pediatric accidental decannulation events: a quality improvement initiative. Int J Pediatr Otorhinolaryngol. (2024) 183:112052. 10.1016/j.ijporl.2024.112052 39106759

[28] Prickett K Deshpande A Paschal H Simon D Hebbar KB. Simulation-based education to improve emergency management skills in caregivers of tracheostomy patients. Int J Pediatr Otorhinolaryngol. (2019) 120:157–61. 10.1016/j.ijporl.2019.01.020 30818130

[29] Van Orne J Brooks M Virgin B DiValerio Gibbs K Ouellette L Acorda DE. Use of simulation in educating caregivers of children with tracheostomies: a scoping review. Respir Care. (2026) 71:784–94. 10.1177/19433654261425222 41873621

[30] Bitners AC Burton WB Yang CJ. Retrospective comparison of Velcro and twill tie outcomes following pediatric tracheotomy. Int J Pediatr Otorhinolaryngol. (2019) 116:192–5. 10.1016/j.ijporl.2018.10.022 30554697

[31] Moser CH Peeler A Long R Schoneboom B Budhathoki C Pelosi PP. Prevention of tracheostomy-related pressure injury: a systematic review and meta-analysis. Am J Crit Care. (2022) 31:499–507. 10.4037/ajcc2022659 36316177

[32] Baker LR Chorney SR. Reducing pediatric tracheostomy wound complications: an evidence-based literature review. Adv Skin Wound Care. (2020) 33:324–8. 10.1097/01.ASW.0000661808.51766.9a 32427789

[33] Al-Samri M Mitchell I Drummond DS Bjornson C. Tracheostomy in children: a population-based experience over 17 years. Pediatr Pulmonol. (2010) 45:487–93. 10.1002/ppul.21206 20425857

[34] Rosenfeld RM Stool SE. Should granulomas be excised in children with long-term tracheotomy? Arch Otolaryngol Head Neck Surg. (1992) 118:1323–7. 10.1001/archotol.1992.01880120049010 1449693

[35] Percul C Lerendegui L Lobos P Liberto D Moldes J Urquizo MM. Association between subglottic stenosis and endotracheal intubation in tracheostomized pediatric patients. Cir Pediatr. (2023) 36:110–5. 10.54847/cp.2023.03.10 37417214

[36] Suresh R Roohani C Wang CS Kou YF Johnson RF Chorney SR. Subglottic stenosis after pediatric tracheostomy. Laryngoscope. (2025) 135:402–8. 10.1002/lary.31736 39189344

[37] Morrison JM Hassan A Kysh L Dudas RA Russell CJ. Diagnosis, management, and outcomes of pediatric tracheostomy-associated infections: a scoping review. Pediatr Pulmonol. (2022) 57:1145–56. 10.1002/ppul.25873 35229491 PMC9313552

[38] Morrison JM Kono N Rush M Hahn A Forster CS Cogen JD. Factors associated with tracheostomy-associated infection treatment: a multicenter observational study. Pediatr Pulmonol. (2024) 59:2761–71. 10.1002/ppul.27117 38860585 PMC11783139

[39] Morrison JM Steuart R Russell CJ. Pediatric tracheostomy-associated respiratory infections: an evolving paradigm. Curr Opin Pediatr. (2026) 38:308–16. 10.1097/MOP.0000000000001559 41944651 PMC13152059

[40] Pearce H Talks BJ Powell J Powell S Brodlie M. A systematic review of antimicrobial therapy in children with tracheostomies. Pediatr Pulmonol. (2024) 59:251–9. 10.1002/ppul.26766 38010838 PMC11497275

[41] McCaleb R Warren RH Willis D Maples HD Bai S O'Brien CE. Description of respiratory microbiology of children with long-term tracheostomies. Respir Care. (2016) 61:447–52. 10.4187/respcare.03518 26670471

[42] Cline JM Woods CR Ervin SE Rubin BK Kirse DJ. Surveillance tracheal aspirate cultures do not reliably predict bacteria cultured at the time of an acute respiratory infection in children with tracheostomy tubes. Chest. (2012) 141:625–31. 10.1378/chest.10-2539 21436240

[43] Russell CJ Mamey MR Koh JY Schrager SM Neely MN Wu S. Length of stay and hospital revisit after bacterial tracheostomy-associated respiratory tract infection hospitalizations. Hosp Pediatr. (2018) 8:72–80. 10.1542/hpeds.2017-0106 29339536 PMC5790296

[44] Amar-Dolan LG Horn MH O’Connell B Parsons SK Roussin CJ Weinstock PH. “This is how hard it is”: family experience of hospital-to-home transition with a tracheostomy. Ann Am Thorac Soc. (2020) 17:860–8. 10.1513/AnnalsATS.201910-780OC 32267725 PMC7328176

[45] DiNoto L Frankel A Wheaton T Smith D Buholtz K Dadiz R. Navigating a new normal: a mixed-methods study of the pediatric tracheostomy parent-caregiver experience. Children. (2025) 12:956. 10.3390/children12070956 40723149 PMC12293189

[46] Aung WT Ong NY Yeo SQC Juhari NSB Kong G Lim N-A. Impact of pediatric tracheostomy on family caregivers’ burden and quality of life: a systematic review and meta-analysis. Front Public Health. (2025) 12:1485544. 10.3389/fpubh.2024.1485544 39886387 PMC11780180

[47] Afolabi-Brown O Marcus M Speciale P Pagala M Kazachkov M. Bronchoscopic and nonbronchoscopic methods of airway culturing in tracheostomized children. Respir Care. (2014) 59:582–7. 10.4187/respcare.02483 24129334

[48] Palumbo KL Smith D Frankel A DiNoto L Wheaton T Buholtz K. Experiences and educational needs of hospital staff providing care to tracheostomy-dependent pediatric patients. Children. (2025) 12:552. 10.3390/children12050552 40426731 PMC12110650

[49] McKeon M Kohn J Munhall D Wells S Blanchette S Santiago R. Association of a multidisciplinary care approach with the quality of care after pediatric tracheostomy. JAMA Otolaryngol Head Neck Surg. (2019) 145:1035–42. 10.1001/jamaoto.2019.2500 31536099 PMC6753653

[50] Mansoor BS Zhang M Chorney S Kou Y-F Wang CS Brooks R. Multidisciplinary tracheostomy teams reduce emergency department utilization in pediatric patients: a retrospective cohort study. Laryngoscope Investig Otolaryngol. (2025) 10:e70327. 10.1002/lio2.70327 41404636 PMC12703805

[51] Brenner MJ Sahay S Silveira RM Moser C Morrison ME Zeitler NK. Addressing education and care gaps in tracheostomy management: insights from a multi-stakeholder global survey. Tracheostomy. (2025) 2:15–28. 10.62905/001c.129226 41607852 PMC12841422

[52] Wells S Shermont H Hockman G Hamilton S Abecassis L Blanchette S. Standardized tracheostomy education across the enterprise. J Pediatr Nurs. (2018) 43:120–6. 10.1016/j.pedn.2018.06.004 30477679

[53] Gaudreau PA Greenlick H Dong T Levy M Hackett A Preciado D. Preventing complications of pediatric tracheostomy through standardized wound care and parent education. JAMA Otolaryngol Head Neck Surg. (2016) 142:966–71. 10.1001/jamaoto.2016.1803 27467686

[54] Sobotka SA Dholakia A Agrawal RK Berry JG Brenner M Graham RJ. Discharge practices for children with home mechanical ventilation across the United States: key-informant perspectives. Ann Am Thorac Soc. (2020) 17:1424–30. 10.1513/AnnalsATS.201912-875OC 32780599 PMC7640721

[55] Beltran-Ale G Simpson R Magruder T Kasi AS Agarwal A Kaslow JA. Discharge practice variability in pediatric chronic home invasive ventilation. Pediatr Pulmonol. (2025) 60:e71130. 10.1002/ppul.71130 40396450 PMC12093446

[56] Neunhoeffer F Miarka-Mauthe C Harnischmacher C Engel J Renk H Michel J. Severe adverse events in children with tracheostomy and home mechanical ventilation: comparison of pediatric home care and a specialized pediatric nursing care facility. Respir Med. (2022) 191:106392. 10.1016/j.rmed.2021.106392 33865662

[57] Verma R Mocanu C Shi J Miller MR Chiang J Wolter NE. Decannulation following tracheostomy in children: a systematic review of decannulation protocols. Pediatr Pulmonol. (2021) 56:2426–43. 10.1002/ppul.25503 34231976

[58] de Miranda LDG Borges LAA Zavaglia LC Mesquita TCL Leite LR Aguiar LT. Decannulation protocol in pediatric patients: case series study. Rev Paul Pediatr. (2025) 43:e2023187. 10.1590/1984-0462/2025/43/2023187 PMC11421412 39319994

[59] Kam K Patzelt R Soenen R. Pediatric tracheostomy speaking valves: a multidisciplinary protocol leads to earlier initial trials. J Child Health Care. (2023) 27:386–94. 10.1177/13674935211070416 35085046

[60] Miles A Wallace S Bax L Keesing M Edwards L Thorpe V. Children with a tracheostomy: global speech-language therapists’ practice. Int J Pediatr Otorhinolaryngol. (2025) 189:112237. 10.1016/j.ijporl.2025.112237 39884020

[61] Grunke C Marshall J Miles A Carrigg B Ward EC. Identifying paediatric populations with increased risk for oropharyngeal dysphagia in acute and critical care settings: a scoping review. Dysphagia. (2025) 40:973–86. 10.1007/s00455-024-10795-y 39704737

[62] Sousa PS Coutinho G Alexandre P Pereira D Spratley J Moura CP. Tracheostomy duration is a key predictor of persistent tracheocutaneous fistulas in children. Cureus. (2025) 17:e78539. 10.7759/cureus.78539 40062117 PMC11887410

[63] Azbell CH Bakeman A McCoy JL Tobey ABJ. Primary versus secondary closure of tracheocutaneous fistula in pediatric patients. Am J Otolaryngol. (2022) 43:103213. 10.1016/j.amjoto.2021.103213 34823915

[64] Saniasiaya J Toll E McCaffer C Neeff M Van Der Meer G Barber C. Management of persistent tracheocutaneous fistula in children: survey among paediatric otorhinolaryngologists. Int J Pediatr Otorhinolaryngol. (2025) 198:112583. 10.1016/j.ijporl.2025.112583 41056646

